# Fluorosed Mouse Ameloblasts Have Increased SATB1 Retention and Gαq Activity

**DOI:** 10.1371/journal.pone.0103994

**Published:** 2014-08-04

**Authors:** Yan Zhang, Ji-Yeon Kim, Orapin Horst, Yukiko Nakano, Li Zhu, Ralf J. Radlanski, Sunita Ho, Pamela K. Den Besten

**Affiliations:** 1 Department of Orofacial Sciences, University of California San Francisco, San Francisco, California, United States of America; 2 Department of Pediatric Dentistry, Dental Research Institute, Pusan National University, Pusan, Korea; 3 Department of Preventive and Restorative Dental Sciences, University of California San Francisco, San Francisco, California, United States of America; 4 Charité - Campus Benjamin Franklin at Freie Universität Berlin, Center for Dental and Craniofacial Sciences, Department of Craniofacial Developmental Biology, Berlin, Germany; Seoul National University, Republic of Korea

## Abstract

Dental fluorosis is characterized by subsurface hypomineralization and increased porosity of enamel, associated with a delay in the removal of enamel matrix proteins. To investigate the effects of fluoride on ameloblasts, A/J mice were given 50 ppm sodium fluoride in drinking water for four weeks, resulting serum fluoride levels of 4.5 µM, a four-fold increase over control mice with no fluoride added to drinking water. MicroCT analyses showed delayed and incomplete mineralization of fluorosed incisor enamel as compared to control enamel. A microarray analysis of secretory and maturation stage ameloblasts microdissected from control and fluorosed mouse incisors showed that genes clustered with *Mmp20* appeared to be less downregulated in maturation stage ameloblasts of fluorosed incisors as compared to control maturation ameloblasts. One of these *Mmp20* co-regulated genes was the global chromatin organizer, special AT-rich sequence-binding protein-1 (SATB1). Immunohistochemical analysis showed increased SATB1 protein present in fluorosed ameloblasts compared to controls. *In vitro*, exposure of human ameloblast-lineage cells to micromolar levels of both NaF and AlF_3_ led to a significantly increase in SATB1 protein content, but not levels of *Satb1* mRNA, suggesting a fluoride-induced mechanism protecting SABT1 from degradation. Consistent with this possibility, we used immunohistochemistry and Western blot to show that fluoride exposed ameloblasts had increased phosphorylated PKCα both *in vivo* and *in vitro*. This kinase is known to phosphorylate SATB1, and phosphorylation is known to protect SATB1 from degradation by caspase-6. In addition, production of cellular diacylglycerol (DAG) was significantly increased in fluorosed ameloblasts, suggesting that the increased phosphorylation of SATB1 may be related to an effect of fluoride to enhance Gαq activity of secretory ameloblasts.

## Introduction

Enamel is the highly mineralized protective outer covering of teeth, conferring strength, abrasion resistance, and protection of the teeth in the oral cavity [1]. Teeth formed in the presence of high levels of systemic fluoride have increased porosity in enamel, referred to as enamel fluorosis [2] [3], [4]. Fluorosed enamel has largely been considered as a defect of enamel maturation, though studies have shown that enamel fluorosis is more severe when fluoride exposure occurs in both secretory and maturation stages of enamel formation, as compared to only in the maturation stage [5].

Ameloblasts form enamel by secreting enamel matrix proteins and directing matrix mineralization [6], . *In vivo* studies of ameloblasts from rodents given high levels of fluoride in drinking water, have shown increased numbers of vacuoles and granules in these cells [9]. Additional cellular effects resulting from ingestion of high levels of fluoride have been reported including increased programmed cell death in the secretory ameloblast zone [10], altered cyclic modulation of ameloblasts between smooth-ended and ruffle-ended cells [11], [12], and impaired endocytosis and intracellular degradation of matrix [13], [14]. *In vitro* studies have shown diverse fluoride-induced effects on ameloblasts that include enhanced proliferation, apoptosis, upregulation of stress related proteins, and elevated F-actin [15], [16], [17], [18], are related to treatment dose, duration and cell type.

It is not yet clear whether fluoride at physiological levels can directly affect ameloblast function, or whether fluorosis results entirely from matrix mediated effects on mineralization [4], [19]. Studies in other tissues have led to the hypothesis that fluoride can mimic the chemical structure of γ-phosphate to bind to GDP, in turn to activate G proteins [20]. To investigate whether fluoride influences activation of G protein in ameloblasts, we used the A/J mouse model, which is highly susceptible to dental fluorosis [21], to determine the potential effects of fluoride on ameloblasts, including Gαq activity.

## Materials and Methods

### Mouse dental fluorosis model

Three-week-old A/J mice were purchased from The Jackson Laboratory (Bar Harbor, ME). Upon arrival, the mice were divided into two groups, half were given deionized water and the other half were given deionized drinking water supplemented with 50 ppm NaF (Sigma-Aldrich, St. Louis, MO) for four weeks.

### Ethics Statement

All animals were housed in the animal facility at University of California, San Francisco. Animals were monitored daily for any change in health status and all treatment and handling followed the approved institutional animal care and use (IACUC) protocol.

### Serum fluoride concentration assessment

After completion of fluoride treatment, the mice were anesthetized with 240 mg/kg tribromoethanol (Sigma-Aldrich, St. Louis, MO). Blood was collected by cardiac puncture and the serum fluoride concentration was determined by an acid microdiffusion technique based on the method previously described by Taves [22], [23]. Briefly, 2 mls of hexamethyldisiloxane presaturated 6 N HCl was placed in a plastic petri dish with a test tube cap glued on it. The test tube cap was filled with 100 µl of 1.65 M NaOH. The petri dish was sealed with a lid with a small hole. The serum sample (500 µl for each sample) was added to HCl through the hole, which was then rapidly sealed with Vaseline and paraffin. Fluoride was allowed to diffuse from the acidified samples and then trap into NaOH for 22 hours. The NaOH and trapped fluoride was dried in 65°C oven, and then was reconstituted to neutral pH with 500 µl of 0.66 M acetic acid. A fluoride ion-specific electrode (Mettler Toledo, Columbus, OH) was used to measure the fluoride concentration of the buffered solution contained in the cap, comparing the measurements of known standards.

### Morphological and histological analyses

Mice were anesthetized with tribromoethanol, and perfuse-fixed with 4% paraformaldyhyde (Sigma-Aldrich, St. Louis, MO). Mandibles were dissected and the incisors were photographed. Three sets of hemimandibles from control and fluoride exposed mice were pre-fixed with 4% paraformaldehyde overnight and then imaged using a Micro XCT-200 (µCT) system (Xradia, Pleasanton, CA). All scans were done at an operating voltage of 90 KVp and 66 µA of current, at an optical magnification 2x. A binning of 2 was used for 3D image reconstruction. All scans were done using the same experimental settings including the distances between specimen, detector, and source. Virtual sections were converted to bmp images using the Xradia TXM3DViewer 1.1.6. software. Comparable images were created using the same intensity thresholds.

Mandibles were otherwise post-fixed in 4% paraformaldehyde for 24 hours, followed by demineralization in 10% EDTA for 4 weeks. Demineralized mandibles were processed and embedded in paraffin and sectioned sagittally at 7 µm thickness and stained with hematoxylin and eosin (H&E).

### Microarray analysis of laser capture microdissected secretory and maturation ameloblasts

Three-week old female A/J mice were given either 0 or 50 ppm sodium fluoride for four weeks, and then perfuse-fixed with 4% paraformaldehyde. Mandibles were dissected from the surrounding soft tissues and post-fixed in 4% paraformaldehyde for 24 hours at 4°C, followed by demineralization in Morse’s solution (10% sodium citrate, 20% formic acid) at 4°C for overnight. The hemimandibles were then embedded in OCT compound, cryo-sectioned at 10 µm thickness. The sections were placed on PEN foil slides (Leica). After staining with hematoxylin & eosin, the slides were placed on the stage of P.A.L.M Laser Dissecting Microscope (Zeiss), and secretory and maturation stage ameloblasts were separately collected by laser microdissection according to cell morphology.

Ameloblasts isolated from 20 sections obtained from 6 mice in either fluoride or control groups were pooled as one sample. Three samples were prepared for each of 4 groups (control secretory ameloblasts, fluorosed secretory ameloblasts, control maturation ameloblasts, fluorosed maturation ameloblasts). The pooled microdissected cells were preserved in lysis buffer including RLT buffer, β-mercaptoethanol and linear acrylamide prior to RNA purification. Total RNA was purified from microdissected cells using QIAGEN RNeasy Micro Kit following the manufacturer’s instruction. Fifty nanograms of each total RNA sample were used for RNA amplification using Ovation Pico WTA System (NuGEN Technologies, San Carlos, CA). Each amplified antisense cDNA product was converted into a sense cDNA transcript (ST-cDNA) and fragmented using WT-Ovation Exon Module (NuGEN Technologies). Fragmented ST-cDNA product was biotinylated using an Encore Biotin Module according to the manufacturer’s instructions (NuGEN Technologies). The biotinylated cDNA was purified using QIAGEN DyeEx 2.0 Spin Kit. The recovered biotinylated cDNA probes were hybridized onto a GeneChip Mouse Gene 1.0 ST Array (Affymetrix, Santa Clara, CA) for 16 hours at 45°C. After hybridization, the array chips were washed, stained with streptavidin phycoerythrin using an Affymetrix Fluidics Station 450 and imaged using an Affymetrix Gene Chip Scanner 3000 (Affymetrix, Santa Clara, CA).

### Gene expression analyses of microarray data

The Robust Multichip Average (RMA) method was used for background adjustment, quantile normalization, and median polish summarization using Affymetrix Expression Console software. The information and data were submitted to Gene Expression Omnibus database, the GEO accession number is GSE57224. Significance Analysis of Microarrays (SAM) was used for statistical analyses of microarray data (http://www-stat.stanford.edu/~tibs/SAM/). Student’s t-test was used for comparing the differential expression of each gene between control and fluoride-treated samples. P<0.05 was considered statistically significant. K-means clustering was used to partition genes with the similar expression pattern. A gene cluster containing *Mmp20* was selected for further analysis, as we have previously showed that MMP20 expression was altered by fluoride in cultured human ameloblast-lineage cells [24].

### Quantitative PCR analysis of SATB1

Total RNA was purified from laser microdissected secretory ameloblasts and maturation ameloblasts using QIAGEN RNeasy Micro Kit. One-hundred nanograms of total RNA from each sample was used as template for cDNA synthesis by using SuperScript III Reverse Transcriptase (Invitrogen, Carlsbad, CA). Primers/probes used to amplify endogenous control 18S and target genes were purchased from Applied Biosystems (Foster City, CA). After standardizing with 18S endogenous control, delta delta CT method [25] was used to quantify the relative expression levels of SATB1 from fluorosed ameloblasts compared to controls.

### SATB1 and PKCα immunochemistry staining

Dissected mandibles were immediately immersed into 4% PFA for 1 day at 4°C followed by decalcification in 8% EDTA (pH 7.3) at 4°C for 4 weeks. The mandibles were then processed through a graded series of ethanol and xylene followed by routine embedding in paraffin and sectioning. The sections were deparaffinized, and blocked for nonspecific staining with 10% swine and 5% goat sera for 1 hour, followed by incubation with rabbit anti-mouse SATB1 antibody (Epitomics, Burlingame, CA) or rabbit anti-phosphorylated PKCα antibody (Abcam, Cambridge, MA) overnight at 4°C. The sections were next thoroughly washed with 0.2% Tween/PBS, and incubated with a biotin-conjugated swine anti-rabbit IgG F(ab’)2 fraction (Dako Cytomation Inc., Carpinteria, CA) for 1 hour at room temperature. The sections were then incubated with alkaline phosphatase conjugated streptavidin (Vector Laboratories Inc., Burlingame, CA) for 30 minutes, and immunoreactivity was visualized using a Vector Red kit (Vector Laboratories Inc.) resulting in pink/red color for positive staining. Counter-staining was done with methyl green (Vector Laboratories Inc). The sections were photographed with a Nikon Eclipse 300 microscope (Compix Inc, Sewickley, PA).

### Cellular diacylglycerol (DAG) analysis

Secretory stage enamel epithelia from 20 control and 20 fluoride-treated mice were microdissected from the mandibular incisors using the molars as reference points [26]. Secretory stage enamel epithelia from five mice receiving the same treatment were pooled together and considered as one sample. Cells were lysed using protease inhibitor cocktail (Sigma-Aldrich) supplemented radioimmunoprecipitation assay (RIPA) buffer (Cell Signaling Technology, Boston, MA). The protein concentration of the cell lysate was determined using the Pierce BCA Protein assay kit. Two-hundred nanograms of cell lysate from each sample was used to determine the cellular DAG concentration using the Mouse DAG ELISA Kit (Cusabio Biotech Co., Newark, DE). Samples and DAG standards provided by kit were added in triplicate to the ELISA plate, which was precoated with anti-mouse DAG capture antibody. After two hours’ incubation at 37°C, the plate was then incubated with a biotin-conjugated anti-mouse DAG detection antibody. Following a wash, the plate was incubated with horseradish peroxidase-avidin for 1 hour at 37°C. TMB (3,3′,5,5′-tetramethylbenzidine) substrate was added to plate to visualize the positive immunoreaction. After stopping the reaction, the plate was read at 450 nm using a Molecular Probe microplate reader (Invitrogen).

### PKC and SATB1 in fluoride treated ameloblast-lineage cells

Human tooth organs were dissected from 16- to 20-week-old aborted and anonymous fetal cadavers under regulations and guidelines set by University of California, San Francisco Committee on Human Research. Tooth organs were first digested with 1.6 mg/ml dispase II (Roche Applied Science, Indianapolis, IN) for 15 minutes at 37°C. Dental epithelial layer were then separated from dental mesenchyme using fine point forceps under a dissecting microscope (Nikon, SMZ1000). Epithelia were digested with 2 mg/ml collagenase/dispase II (Roche Applied Science) at 37°C for 2 hours, followed by a digestion with 0.05% trypsin/EDTA for 5 minutes at 37°C. Keratinocyte growth medium-2 (KGM2) (Lonza) supplemented with 0.05 mM calcium was used to grow ameloblast-lineage cells as previously described [27].

As the first passage of ameloblast-lineage cells reached to 75% confluence, 62.5 µM or 125 µM OAG (1-Oleoyl-2-acetyl-sn-glycerol, Sigma-Aldrich), 10 or 100 µM NaF or AlF_3_ was added to the KGM2 medium. Cells were harvested at a three-hour interval or four-day interval for PKC, p-PKC and SATB1 analyses by Western blot. Briefly, cells were lysed in RIPA buffer (Cell Signaling Technology) supplemented with protease inhibitors. Thirty micrograms of total protein from each sample was resolved on 10% SDS-PAGE gels, and then transferred to PVDF membrane (Millipore, Billerica, MA). After one hour blocking with Odyssey Blocking Buffer (Li-Cor, Lincoln, NE), the membranes were probed with mouse anti-PKC, rabbit anti-p-PKC (Millipore) or rabbit anti-SATB1 antibody (Epitomics, Burlingame, CA) overnight at 4°C. A control blot incubated with rabbit anti-human actin antibody was used to normalize the amount of protein loaded in each sample. The membranes were washed and then probed with IRDye labeled species-specific secondary antibody for 1 hour at room temperature, followed by three washing and scanning on an Odyssey CLx Imaging System (Li-Cor). The intensities of the immunoreactive bands were measured with NIH ImageJ, version 1.46 software.

## Results

### Serum fluoride levels in mice exposed to 50 ppm sodium fluoride increased 4 fold compared with controls

After four weeks’ ingestion of 50 ppm sodium fluoride in drinking water, serum fluoride concentrations of fluoride treated mice (female: 4.48 µM±0.56, n = 5) was significantly increased as compared with the controls drinking deionized water (female: 1.107 µM±0.43, n = 8).

### Incisors of mice exposed to fluoride were morphologically different from the control incisors

**Figure 1:**
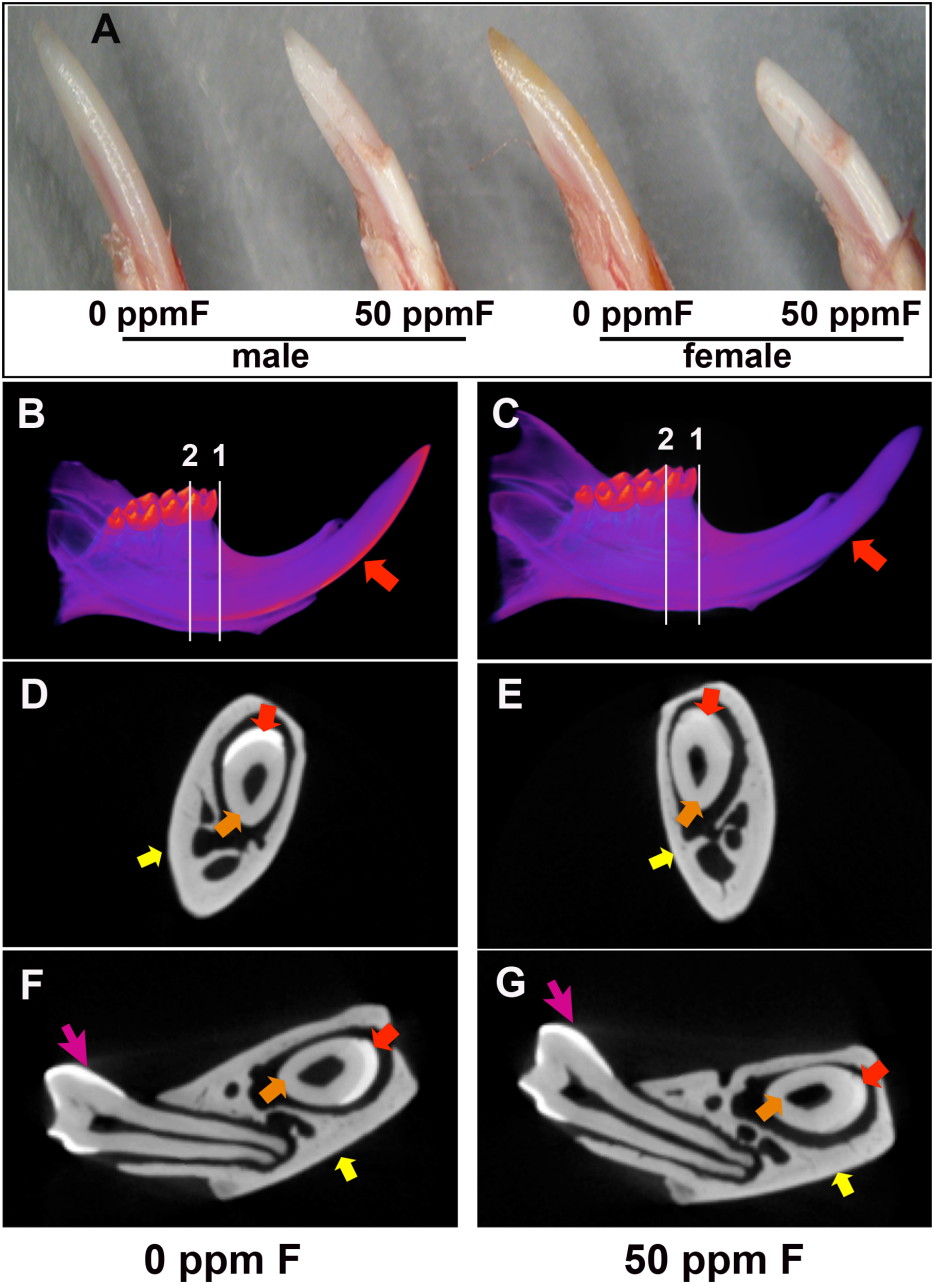
Enamel formation was affected in the incisors of mice ingesting 50 A) Photographs of the erupted portion of incisors from male and female A/J mice, showed whiter appearing easily fractured enamel on fluoride treated incisors as compared to controls. Micro-computed tomography (µCT) analysis of three sets of mouse hemimandibles showed an overall reduction of enamel mineralization in incisors from the fluoride treated mice (C, E, G) as compared to control incisors (B, D, F). B) The 3D reconstructed images of control hemimandibles showed enamel (indicated by red arrow) that was well contrasted from the rest of tissues including dentin and alveolar bone. C) Incisors from the fluorosed mice lacked fully mineralized enamel (indicated by red arrow). Incisor enamel was abraded at the incisal edge. D) MicroCT cross section taken immediately before the location of the first molar showed that incisor enamel (red arrow) was well contrasted from surrounding dentin (orange arrow) and alveolar bone (yellow arrow). E) Cross sections of a fluorosed incisor immediately before the first molar showed enamel (red arrow) had similar x-ray attenuation as the surrounding dentin (orange arrow) and alveolar bone (yellow arrow). Incisor enamel at this plane was obviously less mineralized as compared to the control. F) Cross section across the center of mesial root of first molar in a control incisor showed that enamel (red arrow) was clearly distinguished from dentin (orange arrow) and alveolar bone (yellow arrow). G) At the same plane as panel F, incisor enamel from fluorosed mice had a similar density as dentin and alveolar bone. Fluoride did not affect the enamel of the previously formed molars (pink arrow).

The enamel surface of the erupted portion of incisors of fluoride-exposed mice displayed a chalk-white color, and the distal end of enamel was chipped off, characteristic of fluorosed rodent incisors. The tips of incisors from female fluorosed mice appeared more worn and blunt than those of male fluorosed mice. White spots appeared on the fluorosed incisors, and the enamel on the labial surface of fluorosed incisors appeared striated, mottled and/or pitted (see Fig. 1A).

### MicroCT analysis showed a specific effect of fluoride on mineralization of incisor enamel, with no obvious effect on dentin and bone

The radio opacity of incisor enamel layer in the control hemimandible was significantly higher than that of dentin and alveolar bone. It was evident that mineralization of control incisor enamel rapidly increased from the point perpendicularly aligned with the center of mesial root of the overlaying second molar (see Fig. 1B). Mineralization of fluorosed incisor enamel was reduced, and enamel was lost at the erupted incisor edge (see Fig. 1C). In the cross-sections taken from reconstructed 3-D µCT images at the transition stage of enamel formation, estimated as located immediately before the first molar and under the center of mesial root of the overlying first molar, radio opacity of incisor enamel layer was reduced in fluorotic hemimandible (see Fig. 1E, 1G) as compared to controls (see Fig. 1D, 1F). There was no apparent difference in the radio opacity of enamel layer on control and fluoride-treated molars (see Fig. 1). There was no obvious effect on bone or dentin mineralization, suggesting that the effect of fluoride on enamel formation is more severe at similar fluoride concentrations, than on bone and dentin formation.

### Fluoride treatment altered the morphology of secretory ameloblasts

**Figure 2:**
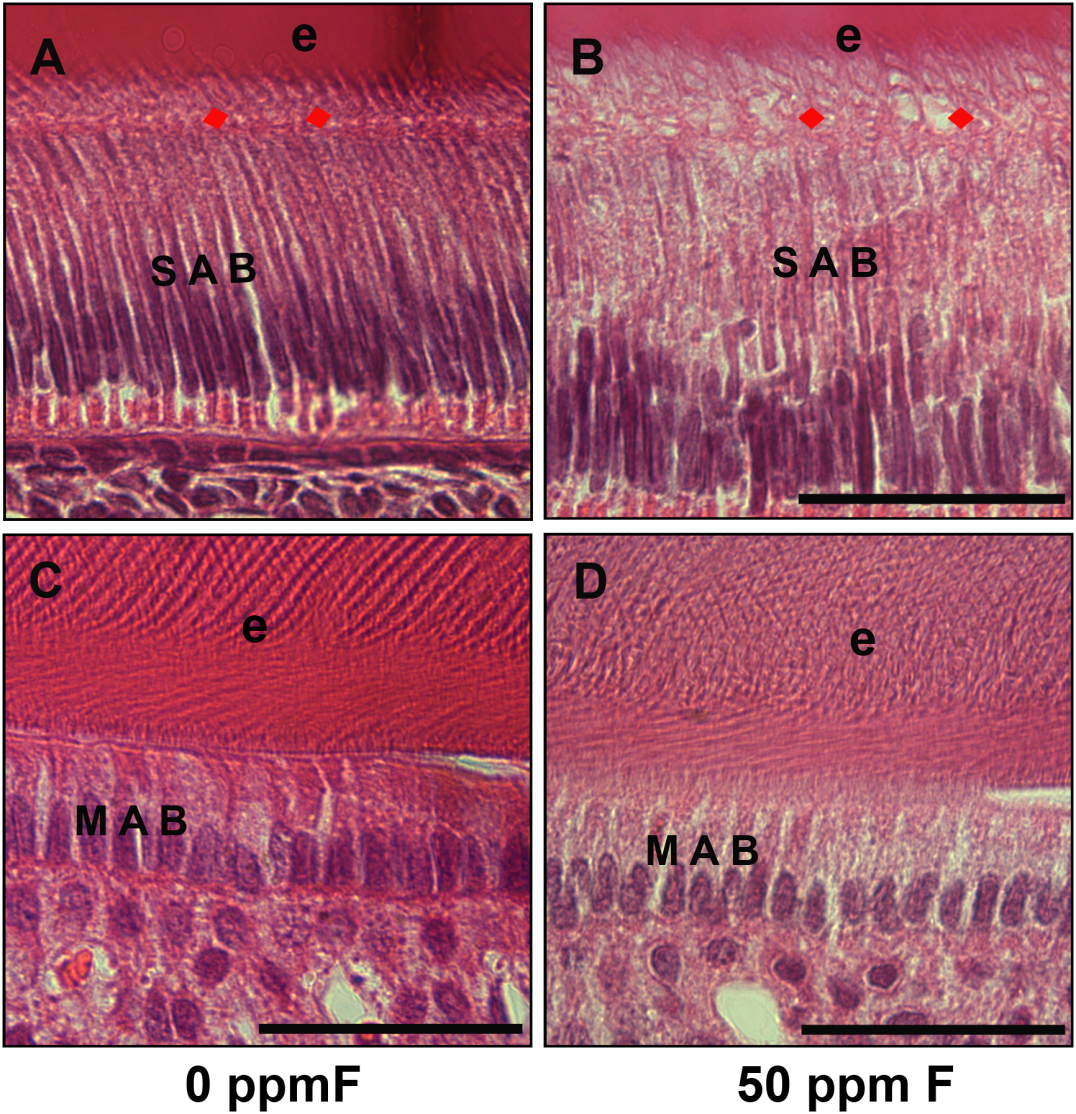
H&E stained histological sections from control and fluoride treated mouse incisors. A) H&E stained control mouse incisors showed elongated and polarized secretory ameloblasts (SAB) and clearly aligned Tomes’ processes (TP) (red arrows). B) Secretory ameloblasts (SAB) in the fluorosed incisors were less organized, had increased amounts of clear vacuoles in cytoplasm. Tomes’ processes (TP) (red arrows) were less distinct as compared to controls. The space between ameloblasts and enamel layer was filled with increased numbers of vacuoles and was obviously wider in the fluorosed incisors. C) H&E stained mouse control maturation ameloblasts (MAB) and mineralizing enamel (e). D) Morphology of the maturation amelobasts (MAB) in the fluorosed mouse incisors showed no obvious differences as compared to controls. Scale bar: 100 µm.

Histological analyses of sagittal sections of incisors showed a somewhat less organized secretory ameloblast layer in the fluorosed incisors (see Fig. 2B) compared to controls (see Fig. 2A). More clear vacuoles accumulated in the cytosol of fluorosed secretory ameloblasts. The nuclei were apparently elongated and less condensed in the secretory ameloblasts of fluorosed incisors (see Fig. 2B). Tomes’ processes of control secretory ameloblasts with “picket-fence” appearance extended and penetrated into the developing organic enamel matrix layer (e) (see Fig. 2A), while Tomes’ processes were less defined in the fluorosed secretory ameloblasts (see Fig. 2B). No obvious morphological changes other than an apparent slight increase in the amount of clear vacuoles were detected in maturation ameloblasts in the fluorosed incisors (see Fig. 2D) as compared to controls (see Fig. 2C).

### Microarray analysis suggested that fluoride delayed the differentiation of secretory ameloblasts toward maturation ameloblasts

**Figure 3:**
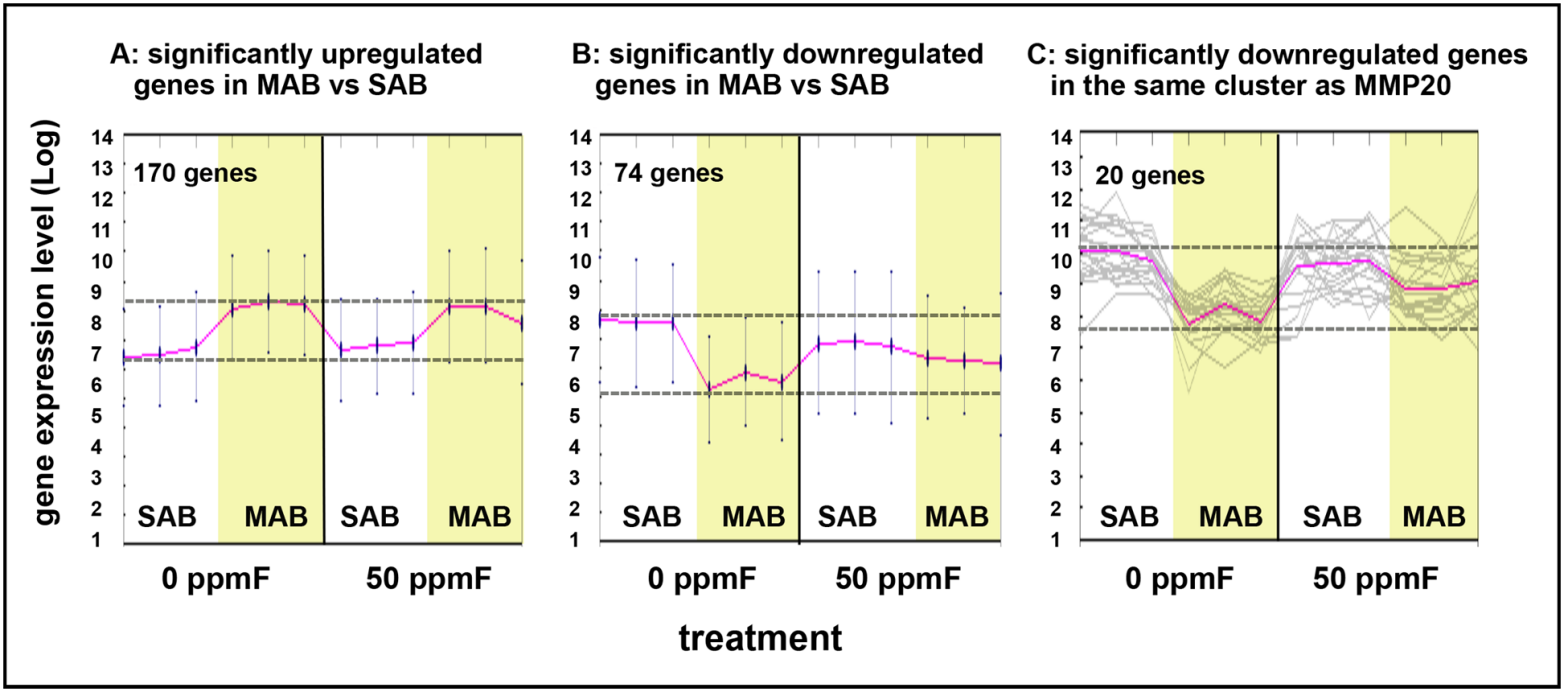
Gene expression patterns in secretory and maturation ameloblasts treated with drinking water supplemented with 50 ppm fluoride for 4 weeks. A) Total 170 genes were significantly upregulated and B) 74 genes were significantly downregulated with at least a two-fold change in maturation ameloblasts (MAB) as compared to secretory ameloblasts (SAB) (n = 3) as analyzed by Significance Analysis of Microarrays (SAM). C) K Means clustering analysis was used to identify genes in the same cluster as *Mmp20* gene. Genes that clustered with *Mmp20* appeared less downregulated from secretory to maturation in fluoride treated as compared to control incisors, though these differences were not statistically significant.

Using the Significance Analysis of Microarrays (SAM) method, we identified 170 genes significantly upregulated (see Fig. 3A) and 74 genes with significantly downregulated (see Fig. 3B) in control maturation ameloblasts compared to control secretory ameloblasts. *Mmp20* mRNA, which is significantly downregulated from secretory to maturation stage ameloblasts [8], was used to perform a K means cluster analysis to identify similarly co-regulated genes. Although the clustered genes were all significantly downregulated from control secretory compared with control maturation ameloblasts, they appeared less downregulated in fluorosed maturation ameloblasts as compared to control maturation ameloblasts (see Fig. 3C). Twenty genes in the same cluster as *Mmp20* have been linked to mechanisms involving calcium homeostasis and extracellular matrix formation, including *Atp2b1*, *Itpr1*, *Anp32e*, *Papln*, *Satb1*, *Lamc2*, and *Plod2.*

Expression of *Satb1*, which is a global transcriptional regulator that regulates the development of epidermis [28], was compared by qPCR using microdissected secretory ameloblasts. As in the microarray, *Satb1* gene expression was reduced 2.8 fold in the control maturation ameloblasts as compared to control secretory ameloblasts (P<0.05 by Student’s t-test). *Satb1* gene expression level was slightly increased in fluoride exposed maturation ameloblasts as compared to control maturation ameloblasts, however these differences were not statistically different.

### SATB1 and phosphorylated PKCα protein were increased in fluorosed ameloblasts as compared to controls

**Figure 4:**
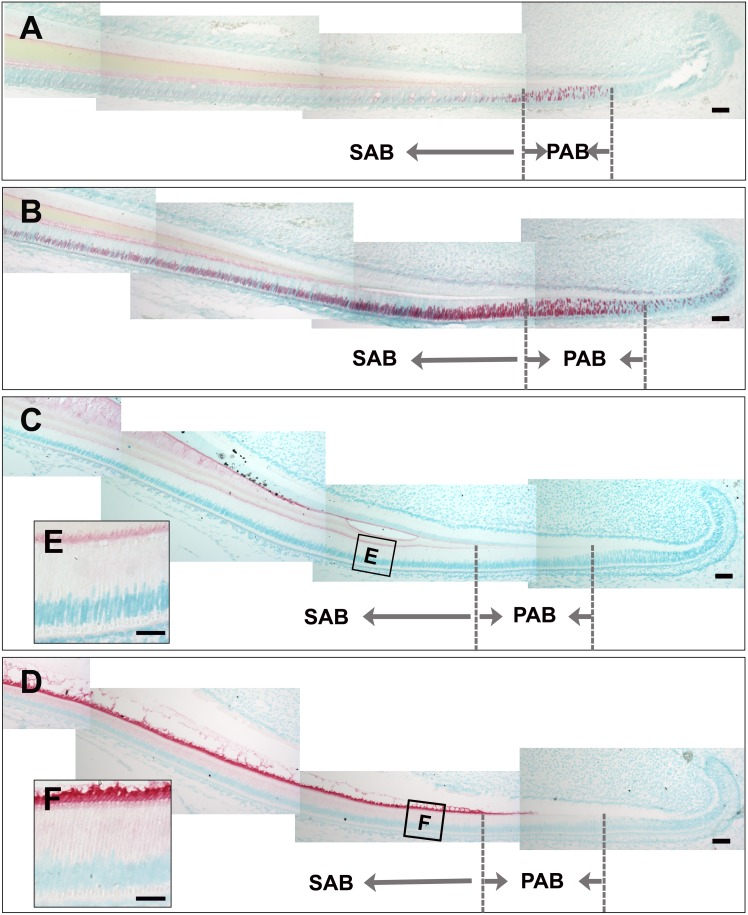
Fluoride treatment resulted in increased SATB1 protein retention and increased phosphorylated protein kinase C alpha (p-PKCα) in ameloblasts. A) Immunostaining of control incisors showed that SATB1 was upregulated in the preameloblalsts (PAB) and downregulated in secretory ameloblasts (SAB). B) Ameloblasts from fluoride treated mice have more SATB1 protein retained in secretory ameloblasts through to the maturation stage. C) Weak immunostaining signal of p-PKCα was detected in the secretory ameloblasts (SAB). D) More intense immunosignal of p-PKC appeared in preameloblasts and continued throughout secretory ameloblasts (SAB). Scale bar: 50 µm.

In control mouse incisors, SATB1 protein was predominately detected in preameloblasts, and was progressively downregulated beginning with secretory ameloblasts (see Fig. 4A). Incisor ameloblasts in fluoride treated mice showed a strong increase in SATB1 protein in preameloblasts, secretory, and maturation ameloblasts (see Fig. 4B). The increased SATB1 protein retention in fluorosed secretory ameloblasts as compared to control secretory ameloblasts was confirmed by Western blot as indicated in Figure S1. Immunostaining of phorphorylated PKCα in ameloblasts was increased at all stages of ameloblast differentiation in fluorosed incisors (see Fig. 4D, 4F) compared to controls (see Fig. 4C, 4E).

### DAG production was increased in fluorosed secretory ameloblasts

**Figure 5:**
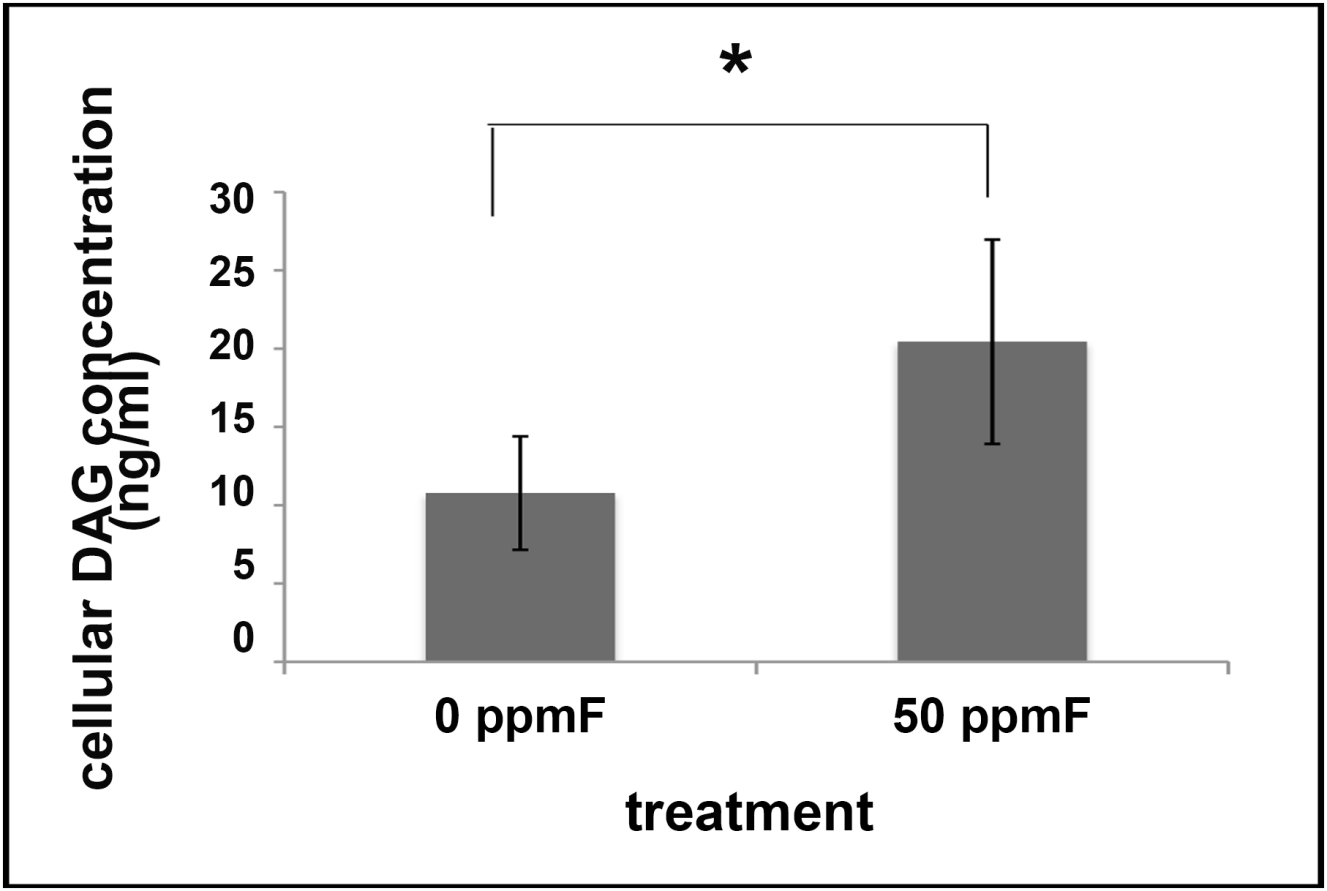
ELISA immunoassay shows mouse cellular DAG significantly upregulated in microdissected secretory ameloblasts from fluoride treated mice as compared to control. *P<0.05 according to student’s unpaired t-test, n = 5.

DAG can activate phosphorylated PKCα [29]. Therefore, consistent with the increased immunostaining for phosphorylated PKCα in fluorosed ameloblasts, we found that the amount of cellular DAG was significantly increased in ameloblasts from fluoride exposed mice, as compared to controls (P<0.05, indicated by Student’s unpaired t-test, see Fig. 5). DAG is the downstream effector of activated Gαq signaling pathway [30], therefore indicating increased Gαq activity in fluorosed ameloblasts.

### Both NaF and AlF_3_ elevated SATB1 protein in cultured human ameloblast-lineage cells

**Figure 6:**
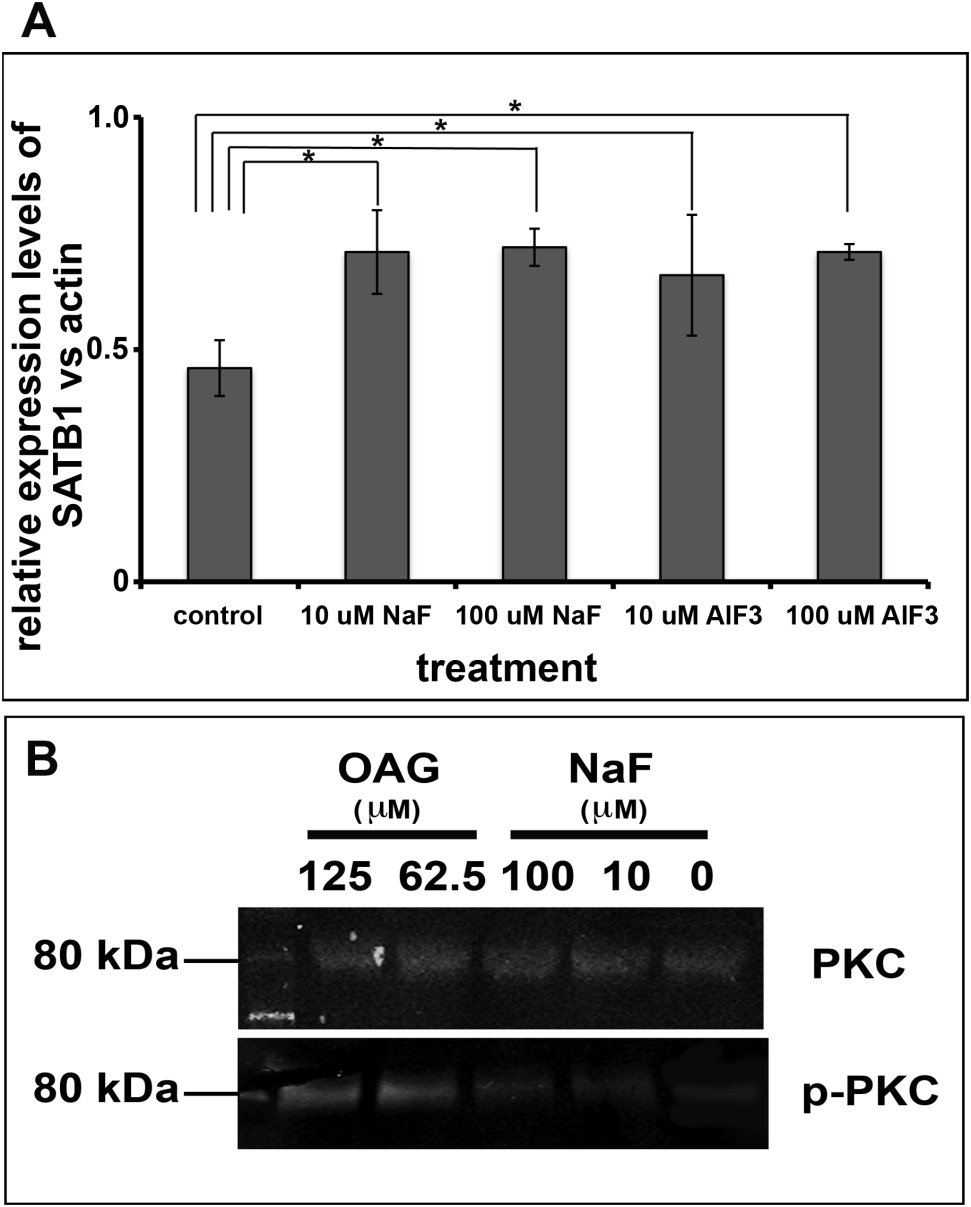
Ameloblast-lineage cells had increased p-PKC and SATB1 when they were exposed to fluoride. A) Densitometry analysis of protein extracted from human ameloblast-lineage cells grown in media supplemented with NaF or AlF_3_ shows significant increase in SABT1 protein when cells were exposed to either 10 or 100 µM fluoride as compared to control. One way ANOVA shows P<0.05, Tukey’s multiple comparison post-test shows * is significantly different from the control samples (n = 4). B) The Western blot analysis showed that OAG, the analog of DAG, at concentration of 62.5 µM or 125 µM significantly increased the phosphorylated PKC, which served as a positive control. NaF at 10 µM or 100 µM also increased the levels of phosphorylated PKC in ameloblast-lineage cells, though at the moderate levels as compared to OAG treated cells. Either OAG or NaF did affect the levels of PKC.

To investigate whether fluoride can directly regulate the abundance of SATB1 protein in cultured cells, we treated primary human fetal ameloblast-lineage cells with 0, 10 µM, or 100 µM NaF or AlF_3_ for four days, and then measured the relative amount of SATB1. Quantitative PCR analysis of SATB1 mRNA collected from cultured cells did not show a significant effect of fluoride on SATB1 gene expression (data not shown). However, densitometric analysis of SATB1 proteins separated and analyzed by Western blot showed that addition of either NaF or AlF_3_ at either 10 µM or 100 µM F significantly elevated SATB1 protein in ameloblast-lineage cells, P<0.05 (see Fig. 6A).

### NaF also elevated phosphorylated PKC in cultured human ameloblast-lineage cells

We further investigated whether NaF influenced the levels of PKC and p-PKC proteins by western blot. This analysis showed that the phosphorylated PKC, but not PKC was significantly increased when ameloblast-lineage cells were exposed to NaF for three hours (see Figure 6B).

## Discussion

Serum fluoride levels of A/J mice given 50 ppm sodium fluoride (1.2 mM) in drinking water, were increased from approximately 1 µM for control mice (0 ppm sodium fluoride in drinking water) to 4.5 µM. Fluoride in the serum of control mice is therefore likely to be a result of the fluoride contained in mouse chow. Similar to the control mouse serum, humans drinking water containing 1 ppm fluoride have approximately 1 µM fluoride in serum [19]. A serum fluoride concentration of 4.5 µM is likely to be found in humans ingesting about 4 ppm fluoride in drinking water. It is not known why rodents require such relatively high levels of fluoride in their drinking water to have serum fluoride levels similar to humans who drink about ten-time less fluoride. However based on our result that the serum levels in this rodent model are biologically relevant to humans, we believe the mechanisms identified in the studies of this dental fluorosis mouse incisor model are relevant to humans.

In the current mouse incisor model, changes in the morphology of secretory stage ameloblasts from fluoride treated mice included increased numbers of clear vacuoles in the cytosol, and less condensed nuclei. Kurebe and colleagues also reported changes in secretory ameloblasts of fluoride treated rats, describing these changes as an accumulation of abnormal large granules in cytoplasm of secretory ameloblasts [31]. Ribeiro and colleagues used ultrastructural morphometric analyses of rat secretory ameloblasts exposed to 100 ppm F, and showed a significant increase (29% increase as compared to control) in volume density of cellular organelles, including rough endoplasmic reticulum, granules, lysosomes, phagic vacuoles, microfilaments and mitochondria [32]. Together, these reports of fluoride related changes in secretory ameloblast morphology suggest that fluoride may interfere with protein metabolism in secretory ameloblasts. How fluoride specifically influences protein synthesis, folding and secretion of secretory ameloblasts needs to be more clearly elucidated.

We investigated the possible effects of fluoride on gene expression using a whole gene transcript microarray to compare relative levels of mRNAs expressed by fluorosed and control ameloblasts. To complete this microarray, we used mRNA isolated from laser microdissected secretory and maturation ameloblasts from incisors of control and fluoride treated adult mice. Though these cells were collected from PFA-fixed tissues, we were able to obtain mRNA with RNA integrity numbers (RIN) ranging from 3 and 6, which was considered adequate to allow a comparative analysis of relative expression levels of whole transcript. The analyses of our microarray data revealed 170 significantly upregulated and 74 significantly downregulated genes in the control maturation as compared to secretory ameloblasts. There were no significant differences in the relative levels of genes expressed by fluorosed secretory or maturation ameloblasts as compared to control ameloblasts at the same stages. However, when we clustered genes that had an expression pattern similar to *Mmp20*, which was significantly downregulated in maturation as compared to secretory ameloblasts, we saw a trend suggesting less downregulation of these genes in fluorosed maturation ameloblasts as compared to control maturation ameloblasts.

One of the potentially affected genes was special AT-rich sequence-binding protein-1 (SATB1). SATB1 is a tissue-restricted chromatin organizer, which regulates gene transcription by attaching chromosomes to nuclear matrix to organize the loop architecture of chromatin, therefore allowing the formation of segregated transcriptional units [33]. Though quantitative PCR analysis using secretory control and fluorosed ameloblasts also did not show significant differences in *Satb1* gene expression, we did find increased and prolonged immunostaining for SATB1 protein in fluorosed ameloblasts. This observation led us to investigate the possibility that fluoride may influence SATB1 post-translational modification.

SATB1 stability is modulated through hydrolysis by caspase-6, following the sumoylation of SATB1 protein at c-terminus [34], [35], [36]. This sumoylation is mediated by SUMO-1, Ubc9 and protein inhibitor of activated STAT (PIAS) family members, which work in concert to enhance SUMO conjugation to SATB1[34]. The sumoylation process is controlled by phosphorylation of SATB1, as PIAS1 interaction with SATB1 is inhibited by phosphorylation of SATB1 at site of T-188 by protein kinase C (PKC) [37]. SATB1 is not only a chromatin organizer, but also functions as a transcription factor by recruiting corepressors histone deacetylases (HDACs) or coactivators histone acetyltransferases (HATs) that directly bind to transcription regulatory regions of target genes [38]. SATB1 functions in a phosphorylated and acetylated status-dependent manner. Therefore we speculate that fluoride-induced retention of SATB1 may result from the increased phosphorylation of SATB1 by activated PKCα. Retained SATB1 may have a role in maintaining a more secretory stage ameloblast phenotype in fluorosed maturation ameloblasts as compared to controls, and also suggested by our microarray.

The possibility that fluoride could affect SATB1 phosphorylation was further supported by our findings of increased PKCα in fluorosed ameloblasts, both *in vivo* and *in vitro*, as well as increased DAG production *in vivo*. DAG is a known activator for PKCα, which could phosphorylate SATB1, in turn potentially stabilizing SATB1 protein. DAG is an effector of activated Gαq, suggesting that fluoride could be enhancing Gαq signaling in secretory stage ameloblasts.

While there are four major types of Gα subunits with differential target proteins in mammals [39]. Our microarray analysis showed that Gαq expression levels were greater in secretory ameloblasts as compared to preameloblasts (data not shown). This predominant Gαq expression in secretory ameloblasts of A/J mice may account for a specific increased phosphorylation of PKC and SATB1 protein at the secretory stage ameloblasts, presumably resulting from fluoride-activated Gαq signaling.

Researchers have long proposed that fluoride may activate G proteins by binding to a metal ion such as aluminum, beryllium or magnesium (XF_n_), and as such mimic the chemical structure of γ-phosphate to bind to GDP, and subsequently affect the activity of phosphoryl transfer enzymes such as GTPase [20]. To determine whether additional metal ions were required for fluoride mediated effects on SATB1 protein retention, possibly linked to increased Gαq activity, we completed additional *in vitro* studies using human primary ameloblast-lineage cells. We found that both AlF_3_ and NaF significantly increased SATB1 protein above that found in control cells, with no difference between these the 2 levels of supplemental fluoride. Furthermore, NaF increased the relative amount of p-PKC. These results indicated that fluoride *in vivo* or *in vitro* can activate Gαq signaling. This may possibly occur by fluoride binding to one of the variety of metal ions widely available in cell culture media, food, or drinking water to mimic γ-phosphate, so that additional metal ions such as AlF_3_ are not required.

**Figure 7:**
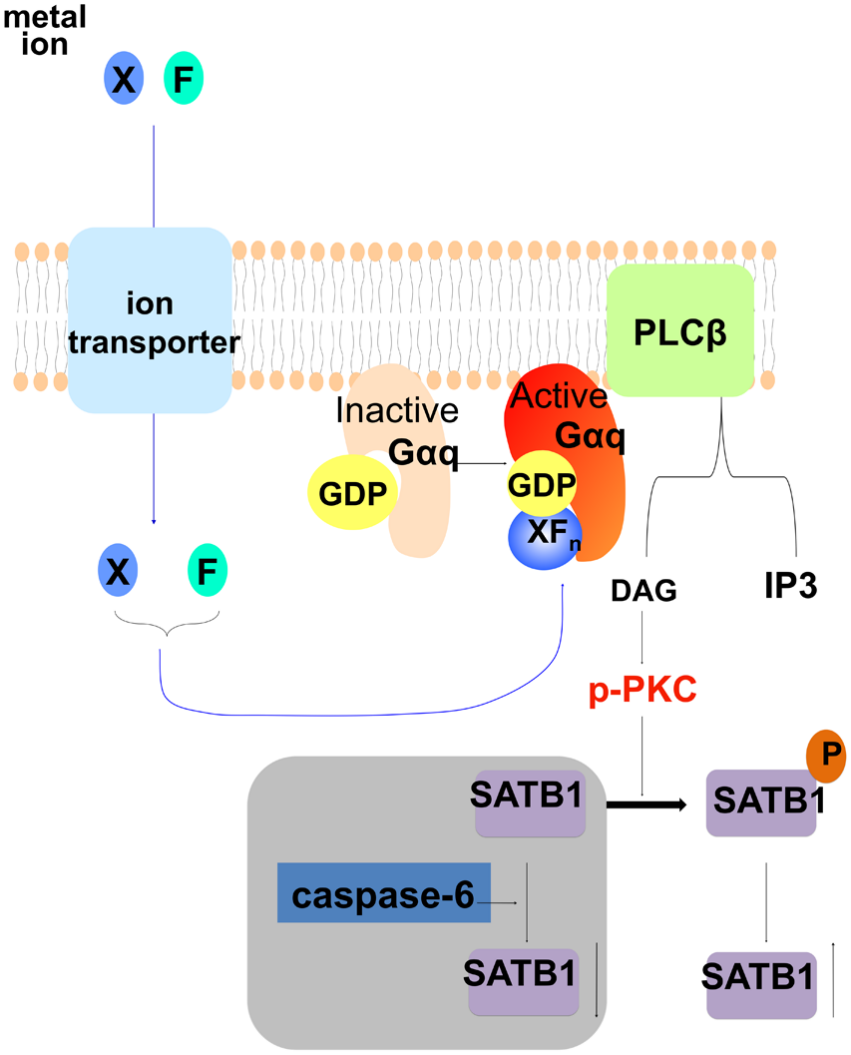
Diagram to elucidate a possible mechanism of how fluoride activates the Gαq signaling pathway, in turn stabilizing SATB1 protein in secretory ameloblasts. This possible mechanism suggests that fluoride can combine with other ions (X) to mimic the chemical structure of γ-phosphate. Upon entering the cells, ion/fluoride complex (XFn) (such as AlF4) can bind to GDP, together to activate Gαq protein in ameloblasts, which in turn activates phospholipase C β (PLCβ) that cleaves phosphatidylinositol 4,5-bisphosphate (PIP_2_) into diacyl glycerol (DAG) and inositol 1,4,5-trisphosphate (IP_3_). Increased DAG sequentially activates PKC, which can then phosphorylate SATB1. SATB1 can be hydrolyzed by caspase-6, while phosphorylation prevents SATB1 from hydrolyzing by caspase-6, resulting in an apparent relative increase in SATB1 in cells.

In summary, these results indicate that fluorosed ameloblasts have higher levels of SATB1 protein, p-PCK and Gαq activity. Gαq signaling, which is known to increase production of DAG, may in turn cause increased levels of phosphorylated PKCα. Increased p-PKCα may enhance phosphorylation of SATB1 to stabilize SATB1 by preventing its degredation in secretory ameloblasts, which could delay ameloblast differentiation (see Fig. 7).

It is interesting to note that Everett and colleagues used linkage analyses to identify QTL intervals on chromosome 11 about 18–51 cM from the centromere, as responsible for the fluoride sensitivity of A/J mice [40]. The mouse *Prkca* gene is located at Chr11∶107933387–108343928 bp (53) where the LOD score is approximately 4.1, potentially implicating a role of PKCα in enamel fluorosis sensitivity. Our data linking stabilized SATB1 to phosphorylated PKC, and its upstream activator DAG, a cellular indicator of activated Gαq signaling, was a finding collected from the A/J mouse strain. Therefore, we are pursuing future studies using other less sensitive mouse strains to determine the possible link between PKC activity and enamel fluorosis susceptibility.

## Supporting Information

Figure S1 Western Blot analysis confirmed that there was more intense signal for SATB1 protein in secretory ameloblasts microdisseted from mandibular incisors of 50 ppm NaF treated mice as compared to control secretory ameloblasts microdissected from mice drinking 0 ppm NaF. Actin served as loading control.
(TIFF)
