# Supplementary figures and images for: Fluorosed Mouse Ameloblasts Have Increased SATB1 Retention and Gαq Activity
Source: PLoS One. 2014 Aug 4;9(8):e103994. doi: 10.1371/journal.pone.0103994 (PMC4121220; doi:10.1371/journal.pone.0103994)

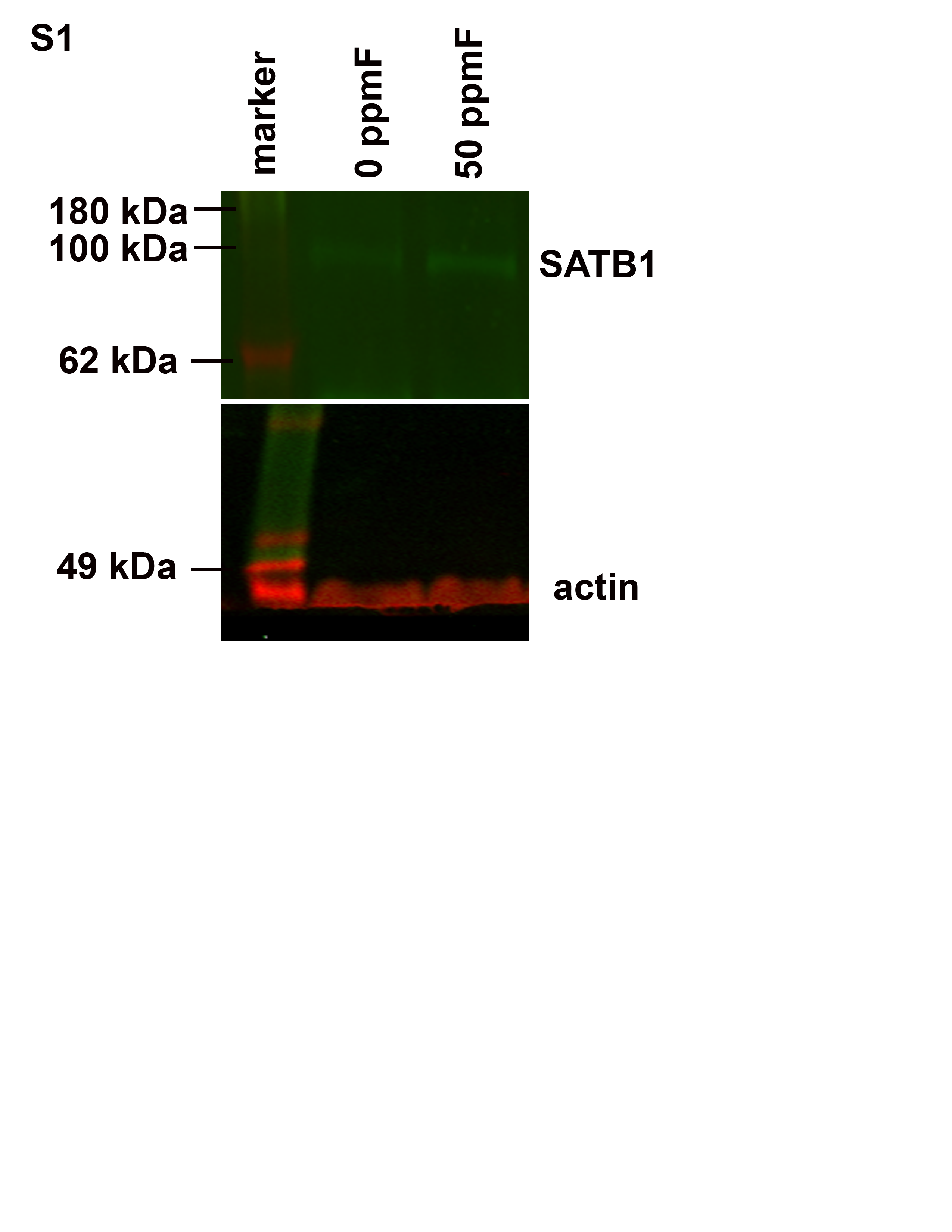

Supplement: Figure S1 — Western Blot analysis confirmed that there was more intense signal for SATB1 protein in secretory ameloblasts microdisseted from mandibular incisors of 50 ppm NaF treated mice as compared to control secretory ameloblasts microdissected from mice drinking 0 ppm NaF. Actin served as loading control. (TIFF) [file pone.0103994.s001.tif]
